# Musculoskeletal Disorders in Patients with Diabetes Mellitus: A Cross-Sectional Study

**DOI:** 10.1155/2018/3839872

**Published:** 2018-06-19

**Authors:** A. Majjad, Y. Errahali, H. Toufik, J. H Djossou, M. A. Ghassem, J. Kasouati, A. El Maghraoui

**Affiliations:** ^1^Rheumatology Department, Mohammed V Military Academic Hospital, Faculty of Medicine and Pharmacy, Mohammed V-Souissi University, Rabat, Morocco; ^2^Endocrinology Department, Mohammed V Military Academic Hospital, Faculty of Medicine and Pharmacy, Mohammed V-Souissi University, Rabat, Morocco; ^3^Department of Statistics, Faculty of Medicine and Pharmacy, Mohammed V-Souissi University, Rabat, Morocco

## Abstract

**Introduction:**

A variety of musculoskeletal disorders (MS) have been associated with diabetes mellitus (DM). This study aimed at assessing the prevalence and associated factors of MS disorders in Moroccan diabetic patients.

**Methods:**

A cross-sectional study enrolled consecutive patients with DM. We recorded demographic features of patients and characteristics of DM. MS disorders and vascular complications were assessed by clinical examinations and investigations. Associated factors of MS disorders were assessed by univariate and multivariate analyses.

**Result:**

376 subjects were included; 84.6% had type 2 DM. The participants' median age was 54 years [45–62]; 41% had one or more vascular complications. 34.4% had one or more MS disorders. Osteoarthritis was present in 19.4% of patients. Hand disorders were seen in 14.4%. Shoulder capsulitis was present in 12.5%. Long duration of diabetes and dyslipidemia were associated with increased prevalence of hand abnormalities (*P* = 0.017; *P* = 0.019, respectively). Age and dyslipidemia were associated with shoulder capsulitis (*P* = 0.019; *P* = 0.047, respectively). Female gender, overweight, and nephropathy were associated with increased odds of osteoarthritis (*P* = 0.009, *P* = 0.004, and *P* = 0.032, respectively).

**Conclusion:**

MS disorders are frequent in this population and associated with various factors. HbA1c level does not appear to be associated with development of MS disorders.

## 1. Introduction

Diabetes mellitus (DM) is a major public health problem worldwide. It was estimated that in 2017 there are 451 million (age: 18–99 years) people with DM. These figures were expected to increase to 693 million by 2045 [1]. A variety of musculoskeletal (MS) disorders have been associated with DM and can cause significant disability [2, 3]. These conditions include shoulder capsulitis (SC), limited joint mobility (LJM), trigger finger (TF), Dupuytren's contracture (DC), Charcot's foot (CF), carpel tunnel syndrome (CTS), osteoarthritis (OA), and other rare complications [4]. The pathophysiology leading to these disorders in patients with DM is not well understood. DM is a chronic metabolic disorder characterized by chronic hyperglycemia. High glucose levels may affect cell function and alter extracellular matrix components of the connective tissue producing damage [3, 5]. In contrast to vascular complications of DM, which has been studied extensively, MS disorders have been hitherto neglected. This study was carried out to investigate the prevalence of MS disorders in Moroccan diabetic patients, their associated factors, and their relationship to other diabetic complications, including micro- and macrovascular complications.

## 2. Materials and Methods

A cross-sectional study was performed between January 2015 and March 2016 and enrolled consecutive patients with type 1 DM (T1DM) and type 2 DM (T2DM) seen in the Endocrinology Department. Patients were included provided they had a history of DM for at least 1 year, diagnosed according to the World Health Organization as a fasting plasma glucose level of ≥126 mg/dL (7.0 mmol/l) [6]. We excluded patients with a history of hand trauma, central or peripheral nervous system disease, chronic rheumatic disease, and end-stage renal disease and patients with thyroid disorders. Ethical approval was obtained from the local ethics committee.

For all patients included in the study, we recorded the following demographic features including age, gender, and body mass index (BMI). We obtained the following clinical information including type and duration of diabetes (in years), antidiabetic treatments, the hemoglobin A1C (HbA1c) levels of the patients, and lipid profile. Dyslipidemia was defined according to American Diabetes Association (ADA) criteria, 2008 [7]. Diabetes was considered controlled if HbA1c level was < 7% according to ADA criteria, 2008 [7]. We also recorded vascular complications of DM, including microvascular complications (retinopathy, nephropathy, and neuropathy), and macrovascular complications (coronary artery disease, peripheral arterial disease, and history of stroke).

MS disorders were assessed using a targeted medical history, standardized physical examination, and investigations if needed. CTS was diagnosed based on clinical symptoms such as pain and sensory disturbances in the thumb, index, middle, and the outer half of the ring fingers and suspected cases were evaluated by electroneuromyography (ENMG). Diagnosis of SC was based on the limitation of active and passive shoulder joint movements in all directions, especially rotational movements. Limited joint mobility was evaluated by the patient “prayer sign”. The diagnosis of DC was based on one or more of the following four features on examination: a palmar or digital nodule, tethering of the palmar or digital skin, a pretendinous band, and a digital flexion contracture. TF was diagnosed by palpating a nodule or thickened flexor tendon with locking happening in extension and flexion of any fingers. OA was researched on patients with chronic joint or back pain. The diagnosis was based on radiographic finding of reduced joint space, subchondral cyst, and osteophytes.

Descriptive statistics were obtained, such as mean values for continuous variables and proportions for categorical variables. The relation between prevalence of MS disorders and various variables was assessed for statistical significance using the Chi-square test. Multiple logistic regression analysis was conducted to assess the multivariate associations between MS disorders and different factors. A *P*-value < 0.05 was considered statistically significant. Statistical analysis was carried out using the Statistical Package for Social Sciences (SPSS, version 23).

## 3. Results

### 3.1. Participant's Characteristics

The study included 376 DM patients. The participants' median age was 54 years [45–62]. There were as many men as women among the participants (*n* = 188, sex ratio=1). 318 patients (84.6%) had T2DM while 58 (15.4%) had T1DM. The median duration of diabetes was 8 years [4-13]. Sixteen percent of our patients had more than 10 years of diabetes. 50.7% were treated with insulin ± oral hypoglycemic. The mean HbA1c value was 8.5 ± 2%. Poor glycemic control was noted in 68.9% of patients. The mean BMI value was 26.4 ± 3.6 kg/m^2^. 45.7% of the patients were overweight, while 15.4% were obese. In this study, 149 patients (39.6%) had dyslipidemia and 155 (41%) had one or more microvascular complications of diabetes. They were dominated by retinopathy in 28.2% of cases, and nephropathy and neuropathy were observed, respectively, in 16.1% and 12.8% of our patients. Sixteen patients (4.3%) had macrovascular complications of DM (coronary artery disease, peripheral arterial disease, and history of stroke) (Table 1).

### 3.2. Prevalence of Abnormalities

The total of patients with MS disorders was 129 (34.3%) out of total 376 cases. They predominated among type 2 diabetics 119 (37.4%) cases versus 10 (17.2%) in type 1 diabetics. Hand disorders were seen in 54 (14.4%) patients; CTS was the most prevalent (8.8%) condition in 33 (8.8%) patients (28 (8.8%) had T2DM versus 5 (8.6%) with T1DM) followed by TF in 22 (5.9%) patients (20 (6.3%) had T2DM versus 2 (3.4%) with T1DM) and LJM in 11 patients, and all of them had T2DM and DC in 2 (0.5%) and all of them had T2DM. SC was present in 47 (12.5%) patients (45 (14.2%) had T2DM versus 2 (3.4%) with T1DM) and CF in 1 case (0.3%) with T1DM. OA was seen in 73 (19.4%) patients (71 (38.4%) had T2DM versus 2 (22.2%) with T1DM) (Table 2). Furthermore, One and two disorders were seen in 25.8% and 5.1% of our patients, respectively, while three disorders in the same patient were seen infrequently (3.5%). The prevalence rates according to demographic and clinical characteristics of patients are shown in Table 3.

In the multivariate analysis, the only factors that were significantly associated with increased prevalence of one or more MS disorders were age above 50 years and dyslipidemia (*P* = 0.002 and *P* = 0.009, respectively). Age above 50 years was an associated factor for SC and OA (*P* = 0.019 and *P* = 0.010, respectively). Duration of diabetes also seemed to confer higher risk; thus patients having diabetes for > 10 years were more likely to have hand disorders (*p* = 0.017). Females were more likely to have OA (*P* = 0.009). Overweight and nephropathy seemed to be significantly associated with OA (*P* = 0.004 and *P* = 0.032, respectively). Dyslipidemia was significantly associated with increased odds of hand disorders and SC (*P* = 0.019 and *P* = 0.047, respectively) (Table 4).

## 4. Discussion

Many studies have evaluated MS disorders in diabetic patients, but most assessed only an individual component, especially upper limb MS abnormalities. Moreover, the relationship between dyslipidemia and MS disorders has been neglected. The current study therefore examined various factors associated with increased prevalence of MS disorders in type 1 and 2 DM including vascular complications and dyslipidemia.

Our study showed three important findings. First, the frequency of MS disorders in DM was 34.4%. The most common MS complications were OA and hand disorders. Second, age above 50 years and dyslipidemia were significantly associated with the development of various MS manifestations. Third, HgA1c level was not found to be linked to any of MS disorders.

MS disorders (one or more) were seen in over one-third of our patients. In previous reports, the prevalence has been reported in about 36–75% of diabetic patients [8, 9]. The lower prevalence of MS complications in our cohort could be related to the fact that we included type 1 and 2 DM. In our study, age above 50 years and dyslipidemia were observed to be associated with such disorders. These findings are similar to the results of previous studies [10, 11].

The prevalence of hand disorders is higher in patients with diabetes compared with nondiabetic subjects [10]. One or more hand disorders were seen in over 14.4% of our patients. In previous reports, the prevalence ranged between 26% and 64% [12, 13]. In our study, CTS was found in 8.8% followed by TF seen in 6.3% and LJM in 3.5% of patients. Duration of diabetes (≥10 years) was observed to be associated with such disorders (*P* = 0.017). In fact, this association between the hand abnormality and the duration of diabetes is a consistent finding [14, 15]. Moreover, we found a significant association between dyslipidemia and increased prevalence of hand disorders (*P* = 0.019).

The association between diabetes and SC is well established [16, 17]. In our study, SC was found in 12.5%. Prior studies reported variable prevalence rates of SC ranging between 11% and 19% in patients with diabetes, compared with 2% to 3% of age-matched controls [18]. In this study, older age and dyslipidemia have been linked to an increased risk of developing SC. Moreover, there was no association between HbA1c level and MS disorders. These findings are similar to the results of some previous studies [10, 19, 20]. Cagliero et al. reported a strong correlation between SC and microangiopathic complications [10]. Nevertheless, such association was not supported by our report.

The most frequent MS disorders in this study were OA, observed in 19.4% of our patients. Most of them had T2DM. This association with T2DM can be partially explained by advanced age and high prevalence of overweight in this population. Hands, spine, and knees are the most common joints affected. We found that older age, female gender, and overweight were significantly associated with the presence of OA (*P* = 0.01, *P* = 0.009, and *P* = 0.004, respectively). These findings were reported in previous study [21, 22]. This relationship is not entirely clear; it has been traditionally attributed to underlying shared risk factors of age and obesity [23, 24]. The coexistence of both OA and DM can be a source of greater disability. In a population cohort with hip and knee OA, Hawker et al. reported that one in six have diabetes. Among those with both OA and diabetes, baseline difficulty walking was a significant predictor of risk for serious diabetes complications [25]. In this study, we found that nephropathy was significantly associated with increased odds of OA (*P* = 0.032).

Finally, this work has some possible limitations. It was an observational study and some additional confounders as smoking and physical activity were not included.

## 5. Conclusion

In the present study, MS disorders occur with a greater frequency in patients with DM. OA was the most frequently seen disorder. One or more MS disorders were significantly associated with various factors, especially dyslipidemia. Blood glucose control does not appear to be associated with development of MS disorders. We suggest that the MS examination should be included in the systematic evaluation of patients with diabetes.

## Figures and Tables

**Table 1:** Descriptive characteristics of the study population (*n* = 376).

		Range
**Age yrs; medians (IQR)**	54 [45-62]	17-88
**Gender**		
**Men n (**%**)**	188 (50)	
**Female n (**%**)**	188 (50)	
**Type of DM**		
**T1DM n (**%**)**	58 (15.4)	
**T2DM n (**%**)**	318 (84.6)	
**HbA1c; means (SD)**	8.5±1.9	4.2-17
**BMI kg/m** ^**2**^ **; means (SD)**	26.4±3.6	17-42
**Duration of DM yrs; medians (IQR)**	8 [4-13]	1-37
**Retinopathy n (**%**)**	106 (28.2)	
**Nephropathy n (**%**)**	61 (16.1)	
**Neuropathy n (**%**)**	48 (12.8)	
**Macrovascular complications n (**%**)**	16 (4.3)	
**Dyslipidemia n (**%**)**	145 (41)	

DM: diabetes mellitus; T2DM: type 2 diabetes mellitus; T1DM: type 1 diabetes mellitus; HbA1c: hemoglobin A1c; BMI: body mass index.

**Table 2:** Prevalence and distribution of cases according to MS disorders in relation to type of diabetes.

**MS disorders**	**Type of diabetes**						
	Type 1		Type 2		*P*	**Total** **N =376**	
	**N = 58**		**N = 318**				
	*n*	%	*n*	%		*n*	%
**Osteoarthritis**	2	3.4	71	22.3	**0.001** ^**∗**^	73	19.4
**Shoulder capsulitis**	2	3.4	45	14.2	**0.023** ^**∗**^	47	12.5
**Carpel Tunnel Syndrome**	5	8.6	28	8.8	0.964	33	8.8
**Limited joint mobility**	0	0	11	3.5	0.151	11	2.9
**Trigger Finger**	2	3.4	20	6.3	0.397	22	5.9
**Dupuytren's contracture**	0	0	2	0.6	0.545	2	0.5
**Total MS disorders**	10	17.2	119	37.4	0.149	129	34.4

MS: musculoskeletal.

**Table 3:** Prevalence of MS disorders according to sociodemographics, vascular complications, and other relevant variables in diabetic patients.

	Hand disorders	Shoulder Capsulitis	Osteoarthritis	MS disorders (total)
	*n* (%)	*n* (%)	*n* (%)	*n* (%)
Age (yrs)				
<50	13(9.8)	6(4.5)	9(6.8)	23(17.3)
≥50	49(16.9)	41(16.9)^*∗*^	64(26.3)^*∗*^	106(43.6)^*∗*^
Gender				
Male	22 (11.7)	22(11.7)	28(14.9)	58(30.9)
Female	32 (17)	25 (13.3)	45(23.9)^*∗*^	71(37.8)
BMI (%)				
Normal	15 (10.3)	15(10.3)	15(10.3)	38(26)
overweight	30(17.4)	20(11.6)	39(22.7)^*∗*^	65(37.8)^*∗*^
obesity	9(15.5)	12(20.7)	19(32.8)	26(44.8)^*∗*^
Type DM				
T1DM	7(12.1)	2(3.4)	2(3.4)	10(17.2)
T2DM	47(14.8)	45(14.2)^*∗*^	71(22.3)^*∗*^	119(37.4)^*∗*^
Duration (yrs)				
<10	20(9.9)	23(11.4)	29(14.4)	53(26.2)
≥10	34(19.5)^*∗*^	24(13.8)	44(25.3)^*∗*^	76(43.7)^*∗*^
HbA1c (%)				
< 7	6(7.2)	9(10.8)	11(13.3)	18(21.7)
≥ 7	48(16.4)^*∗*^	38(13)	62(21.2)	111(37.9)^*∗*^
dyslipidemia				
Yes	30(20.1)^*∗*^	27(18.1)^*∗*^	42(28.2)^*∗*^	70(47)^*∗*^
Non	24 (10.6)	20(8.8)	31(13.7)	59(26)
Retinopathy				
Yes	16 (15.1)	15(14.2)	31(29.2)^*∗*^	49(46.2)^*∗*^
Non	38 (14.1)	32(11.9)	42(15.6)	80(29.6)
Nephropathy				
Yes	8(13.1)	11(18)	21(34.4)^*∗*^	28(45.9)^*∗*^
Non	46(14.6)	36(11.4)	52(16.5)	101(32.1)
Neuropathy				
Yes	11(22.9)	6(12.5)	11(22.9)	24(50)^*∗*^
Non	43(13.1)	41(12.5)	62(18.9)	105(32)
Macrovascular disorder				
Yes	3(18.8)	6(37.5)	8(50)	13(81.3)^*∗*^
Non	51(14.2)	41(11.4)	65(18.1)	116(32.2)

^*∗*^ *P*<0.05; 95 % CI

DM: diabetes mellitus; MS: musculoskeletal; T2DM: type 2 diabetes mellitus; T1DM: type 1 diabetes mellitus; HbA1c: hemoglobin A1c; BMI: body mass index.

**Table 4:** Multivariate analysis of factors associated with MS disorders.

variable	Hand disorders		Shoulder capsulitis		Osteoarthritis		MS disorders (total)	
	OR (95% CI)	*P value*	OR (95% CI)	*P value*	OR (95% CI)	*P value*	OR (95% CI)	*P value*
Age (yrs)								
<50			1		1		1	
≥50			3.06	0.019	3.15	0.010	2.94	0.002
			(1.09-8.62)		(1.31-7.54)		(1.50-5.73)	
Gender								
Male					1			
Female					2.15	0.009		
					(1.20-3.85)			
BMI (%)								
Normal					1			
overweight					1.84	0.004		
					(1.21-2.80)			
obesity					0.6	0.128		
					(0.3-1.15)			
Duration (yrs)								
<10	1							
≥10	2.09	0.017						
	(1.14-3.83)							
Dyslipidemia								
Non	1		1				1	
Yes	2.03	0.019	1.90	0.047			1.89	0.009
	(1.12-3.68)		(1-3.59)				(1.17-3.06)	
Nephropathy								
Non					1			
Yes					2.14	0.032		
					(1.06-4.29)			

BMI: body mass index.

## Data Availability

The datasets generated during and/or analyzed during the current study are available from the corresponding author on reasonable request.
